# Decitabine as epigenetic priming with CLAG induce improved outcome of relapsed or refractory acute myeloid leukemia in children

**DOI:** 10.1186/s13148-024-01677-z

**Published:** 2024-05-09

**Authors:** Na Zhang, Hong Li, Dan Wang, Zhen Wang, Jia-Shi Zhu, Kai Chen, Hui Jiang, Jing-Bo Shao, Cheng Cai

**Affiliations:** 1 Department of Hematology and Oncology, Shanghai Children’s Hospital, School of Medicine, Shanghai Jiao Tong University, No. 1400, West Beijing Road, Shanghai, 200040 China; 2 Department of Neonatology, Shanghai Children’s Hospital, School of Medicine, Shanghai Jiao Tong University, No. 355, Luding Road, Shanghai, 200062 China

**Keywords:** Acute myeloid leukemia, Children, Cladribine, Decitabine, Refractory, Relapsed

## Abstract

**Background:**

Decitabine (DAC), a DNA methyltransferase inhibitor, has shown efficacy combined with chemotherapy for relapsed or refractory (R/R) acute myeloid leukemia (AML) in adults, but less is known about its efficacy in children. Accordingly, we conducted a study which involved a priming regimen consisting of DAC with cladribine, cytarabine, and granulocyte-stimulating factor (DAC-CLAG) and compared the efficacy and safety of this regimen with CLAG alone.

**Methods:**

A total of 39 R/R AML children who received the CLAG or DAC-CLAG regimen in Shanghai Children’s Hospital were retrospectively enrolled in this non-randomized study. These regimens were studied sequentially over time. Twenty-two patients received CLAG from 2015, while 17 patients were administered epigenetic priming with DAC before CLAG from 2020. Patients were subsequently bridged to stem cell transplantation (SCT) or consolidation chemotherapy. Complete remission (CR) and adverse effects were analyzed by Fisher’s exact test, and survival was analyzed by the Kaplan–Meier method.

**Results:**

DAC-CLAG conferred a numerically higher CR compared to CLAG (70.59% vs 63.64%; *P* = 0.740). High CR rates occurred in patients with good cytogenetics (*P* = 0.029) and prior induction without cladribine (*P* = 0.099). The 1-year event-free survival (EFS) was 64.71% ± 11.59% and 63.31% ± 10.35% in the DAC-CLAG and CLAG group (*P* = 0.595), and 1-year overall survival (OS) was 81.45% ± 9.72% and 77.01% ± 9.04%, respectively (*P* = 0.265). The 1-year OS and EFS after SCT were higher in the DAC-CLAG than in the CLAG cohort (100% *vs* 92.31% ± 7.39%,* P* = 0.072; 92.31% ± 7.39% vs 85.71% ± 9.35%, *P* = 0.158). Univariate analysis revealed that a good prognosis included good cytogenetics (*P* = 0.002), non-complex karyotype (*P* = 0.056), CR on reinduction (*P* < 0.0001), and bridging to SCT (*P* = 0.0007). Use of a hypomethylating agent (*P* = 0.049) and bridging to SCT (*P* = 0.011) were independent prognostic factors. Grade 3/4 hematologic toxicity and infection were the main adverse events.

**Conclusions:**

DAC prior to the CLAG regimen improved remission in pediatric R/R AML, and was feasible and well tolerated. CLAG ± DAC as a salvage therapy prior to SCT induced improved survival.

**Supplementary Information:**

The online version contains supplementary material available at 10.1186/s13148-024-01677-z.

## Introduction

The outcome of relapsed or refractory (R/R) acute myeloid leukemia (AML) in children remains poor, and the optimal salvage regimen is uncertain. Conventional chemotherapy with anthracycline- and cytarabine-based regimens are disappointing with complete remission (CR) of 22–23% in children [1, 2]. There is currently no standard reinduction or salvage chemotherapy for R/R AML, and many trials are focused on adding targeted therapies to AML regimens. Induction regimens integrating cladribine have shown promising outcomes in R/R AML. Regimens including cladribine, cytarabine, and granulocyte-stimulating factor (CLAG) resulted in excellent outcomes with a 50–62.7% CR rate in adult R/R AML [3–5] and 66.7% in children [1]. According to reported studies, the addition of medications with different mechanisms to CLAG can increase the efficacy for R/R AML. CLAG regimens plus “X” includes mitoxantrone (M) [2, 6], pegylated liposomal doxorubicin [7], or aclacinomycin [8], most of which are cytotoxic agents. The CR rates due to the above regimens range from 49 to 75%, which is comparable to the CLAG regimen.

In recent years, hypomethylating agents (HMAs) have shown good tolerance in AML patients and these HMAs-combination regimens are potentially efficacious in AML [9]. Studies have shown that DNA hypermethylation is negatively correlated with the efficacy of induction chemotherapy in AML [10]. Compared to newly diagnosed AML patients, there is an increase in the frequency and density of DNA methylation in relapsed patients [11]. Cladribine was also shown to induce DNA hypomethylation by inhibiting S-adenosylhomocysteine hydrolase, which was distinct from traditional DNA methyltransferase inhibitors [12]. Alternating cladribine with a HMA such as decitabine (DAC) in a protocol may be complementary and overcome potential resistance.

Cladribine-based regimens have shown efficacy in AML, and HMAs may be potentially efficacious in R/R AML, but little is known about the efficacy of these regimens in children. Considering the above-mentioned challenges in the treatment of R/R AML, we conducted the “priming” use of DAC and a sequential modified CLAG regimen in children. The efficacy and tolerability of DAC-CLAG were determined compared with the CLAG regimen. In addition, the probable synergism of incorporating a HMA and nucleoside analogs was determined by superior response rates translating into better long-term survival.

## Patients and methods

### Patients

In this retrospective study, 39 patients with R/R AML treated at Shanghai Children’s Hospital (Shanghai, China) were enrolled. The inclusion criteria for this study were patients with R/R AML and age < 18 years. The definition of R/R AML was previously described [1]. The exclusion criteria were as follows: (a) patients with acute promyelocytic leukemia; (b) patients with secondary AML, such as AML secondary to Fanconi anemia, or Shwachman syndrome; and (c) patients with severe infection, and cardiac, liver and kidney insufficiency who had an expected survival time less than 3 months. Patient enrollment was from July 2015 to July 2023 on two clinical trials: CLAG regimens for patients with R/R AML approved by the Ethics Committee of Shanghai Children’s Hospital (No. 2015035); DAC-CLAG regimens for patients with R/R AML registered at chictr.org.cn (No. ChiCTR2100045432). The studies were conducted in accordance with the Declaration of Helsinki and were approved by the local institutional review boards. The follow-up period was 21 months (1–99) in the CLAG group and 13 months (1–40) in the DAC-CLAG group.

### Study design

In this non-randomized research, patients were treated with one of two reinduction regimens at the single center of the division of Hematology and Oncology, Shanghai Children’s Hospital. These regimens were studied sequentially over time. Reinduction with the CLAG regimen started in July 2015 and comprised cladribine (9 mg/m^2^, max 10 mg; as a 3-h infusion on days 1–5), cytarabine (200–400 mg/m^2^/day; 21-h infusion on days 1–5), and G-CSF (5 μg/kg, max 300 μg; on days 0–5, omitted if the leukocyte count was above 20 × 10^9^/L). Reinduction with the DAC-CLAG regimen started in May 2020 and comprised DAC (20 mg/m^2^, 1-h on days − 1 to − 5) and subsequently the CLAG regimen as described above (cytarabine at 200 mg/m^2^/day in the DAC-CLAG regimen). Each course was repeated every 4–6 weeks when meeting the chemotherapy standard (absolute neutrophil count ≥ 1 × 10^9^/L and platelet count ≥ 80 × 10^9^/L) and resolution of non-hematologic toxicities to ≤ Grade 1. If patients achieved CR after one course, they were given another one or two courses for consolidation, or continuing with SCT. If patients had no remission (NR) after one course and NR/partial remission (PR) after two courses, they were withdrawn from the study.

Risk stratification was evaluated according to Diagnosis and management of AML 2017 recommendations on European LeukemiaNet (ELN) [13]. Karyotype or molecular abnormalities were summarized as good risk [t(8;21), inv(16) or t(16;16), NPM1mut without FLT3-ITD or with FLT3-ITD^low^, CEBPA double mut], poor risk (DEK-NUP214, BCR-ABL1, complex karyotype abnormalities, 5 or 7 monosomy, 5q or 7q abnormalities, -17/abnormal(17p), 11q23 abnormalities except t(9;11), FLT3-ITD^high^, ASXL1, mutated TP53), or intermediate risk (karyotype or molecular abnormalities not encompassed by the good or poor risk groups).

### Efficacy evaluation

Bone marrow (BM) examinations were performed on day 28 of each course. Efficacy was evaluated according to the following criteria [1]: CR, blast cell levels in the BM < 5%, platelet levels ≥ 100 × 10^9^/L, neutrophil count ≥ 1.0 × 10^9^ /L, and absence of extramedullary infiltration; CR with incomplete hematopoietic recovery (CRi) was defined the same as CR, but without recovery of either platelet count or neutrophil count to the above levels; PR, blast cell levels in the BM 5–25%; NR, other than CR or PR. Minimal residual disease (MRD) was assessed using multi-parameter flow cytometry (FCM) validated to a sensitivity level of 0.01% from May 2020, available in 19 patients. MRD positive was defined as > 0.01% of cells with an aberrant leukemia-associated immune phenotype. BM relapse was defined by the presence of > 5% of leukemic cells in BM. Molecular MRD was assessed by reverse transcription-polymerase chain reaction (RT-PCR) or next-generation sequencing (NGS).

### Safety evaluation

Hematologic toxicity, infection, and organ dysfunction were recorded. Toxicity classification was performed according to National Cancer Institute Common Toxicity Criteria version 4.0.

### Cytogenetic and molecular exploration

All patients underwent morphology, immunology, molecular and cytogenetic (MICM) examination of BM. Morphology was classified according to French-American-British (FAB) classification, and aberrant immunophenotype was detected using multi-parameter FCM. Conventional cytogenetic testing was performed on baseline BM samples using G-banded analysis. The results were reported using the International System for Human Cytogenetic Nomenclature. Molecular testing for fusion genes included RT-PCR (43–66 genes) and fluorescence in situ hybridization analysis. The molecular abnormalities of mutated genes were detected by NGS. The next-generation RNA sequencing assay started in May 2020.

### Statistical analysis

The quantitative data with normal distribution were expressed as the mean ± standard deviation (SD) and were compared using the t test. Non-normally distributed data were presented as median and full range, using the Mann–Whitney test for two groups. Frequency and percentage were presented for categorical variables, using Fisher’s exact test or the chi-square (*χ*^2^) test. Overall survival (OS) was defined as the time from induction administration to death due to any cause or censored at last follow-up. Event-free survival (EFS) was defined as the time from the start of therapy to the date of primary refractory disease, relapse or death from any cause or the last follow-up date. Patients who were lost to follow-up were censored at the last date when they were known to be alive. OS and EFS were estimated by the Kaplan–Meier method, and the curves of the two cohorts were compared using the log rank test. The Cox proportional hazards model was used for univariate and multivariate analyses. Statistical analyses were performed with GraphPad Prism 9.5 and SPSS 19.0. All reported *P* values are two-sided, and a significance level of *α* = 0.05 was used.

## Results

### Patient characteristics

**Fig. 1:**
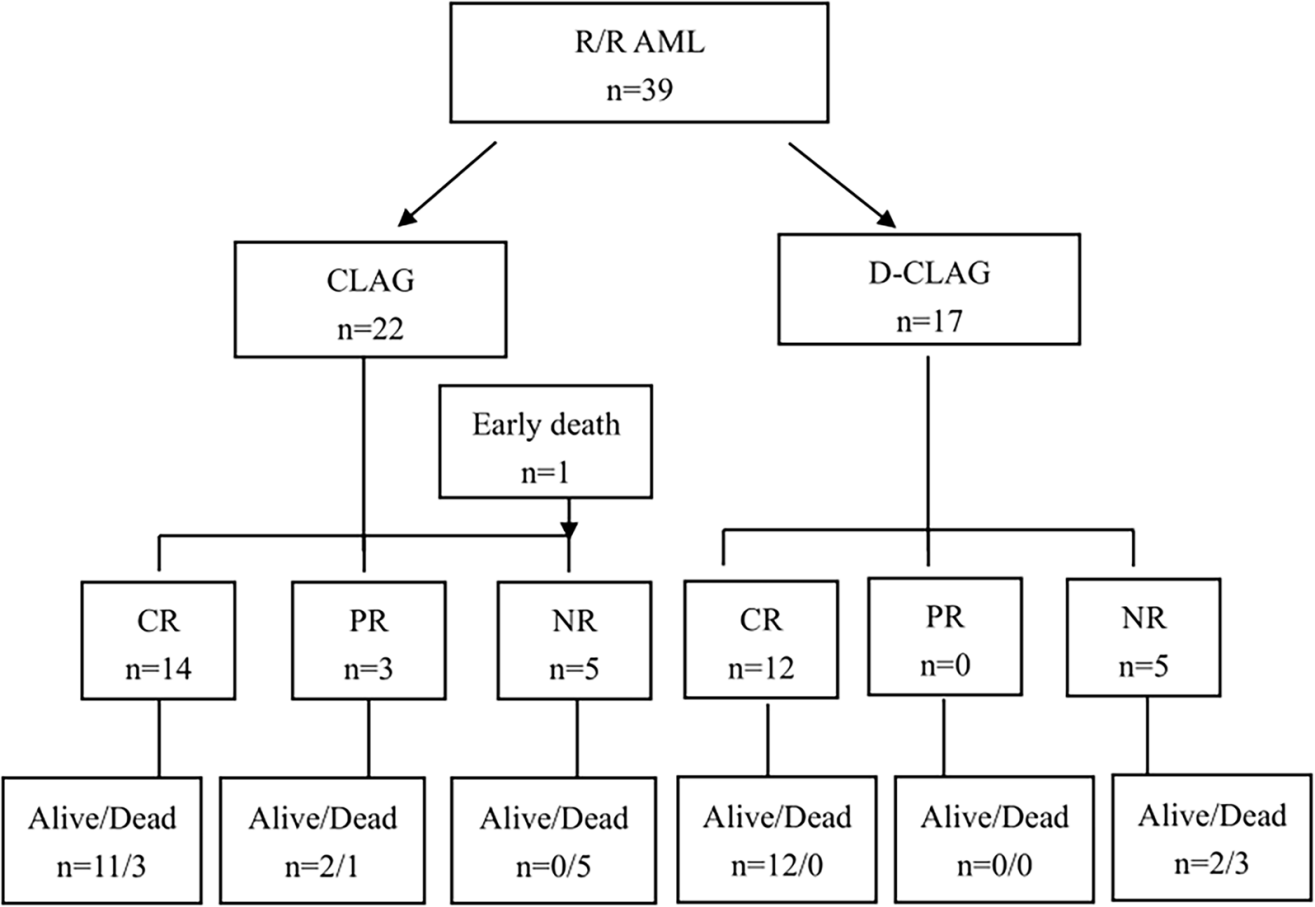
Screening diagram of 39 pediatric patients with relapsed/refractory acute myeloid leukemia

Twenty-two patients were enrolled in the CLAG cohort and 17 patients were enrolled in the DAC-CLAG cohort. The mean age of patients in the CLAG cohort was 102.5 ± 60.7 months and was 81.4 ± 59.4 months in the DAC-CLAG cohort. The majority of patients were diagnosed with M2 (*n* = 12, 30.77%), M4 (*n* = 11, 28.21%) and M5 (*n* = 12, 30.77%). Four (10.26%) patients had prior allogeneic SCT (allo-SCT) before the salvage treatment. Three patients were sequentially enrolled at the primary refractory and relapsed phases. Among these three patients, one with primary refractory disease achieved CR after two courses of CLAG with a remission period of 5 months. This patient had NR with the CLAG regimen at the relapsed phase. Another patient with refractory disease achieved CR following the CLAG regimen. However, the DAC-CLAG regimen was ineffective at the relapsed phase after allo-SCT. The third patient still had refractory disease after CLAG, and treatment with DAC-CLAG was ineffective. The median follow-up time was 18 (1–99) months. There was no difference in baseline characteristics between the two cohorts (Table 1). The last follow-up day was December 31, 2023. The outcomes of the patients in the two cohorts are shown in Fig. 1.

**Table 1:** R/R AML patient characteristics on reinduction with the CLAG regimen with or without decitabine

Features	Total, *n*	CLAG, *n*	DAC-CLAG, *n*	*P* value
Age, months	93.3 ± 60.3	102.5 ± 60.7	81.4 ± 59.4	0.283
Gender, F/M	9/30	5/17	4/13	1.000
FAB classification				0.800^a^
M0	1	0	1	
M2	12	7	5	
M4	11	7	4	
M5	12	6	6	
M7	1	0	1	
Myeloid sarcoma	2	2	0	
Disease status				0.753
Relapse	15	9	6	
Refractory	24	13	11	
Relapse number				1.000
1	20	13	7	
2–3	2	2	0	
Cytogenetic risk				0.318^b^
Favorable	2	0	2	
Intermediate	11	9	2	
Adverse	26	13	13	

*CLAG* cladribine, cytarabine, and granulocyte colony-stimulating factor, *DAC* decitabine, *n* number, *FAB classification* French-American-British classification, *F/M* female/male, *R/R AML* relapsed or refractory acute myeloid leukemia

^a^Analysis with M2, M4, M5

^b^Favorable and intermediate were combined as one column

**Fig. 2:**
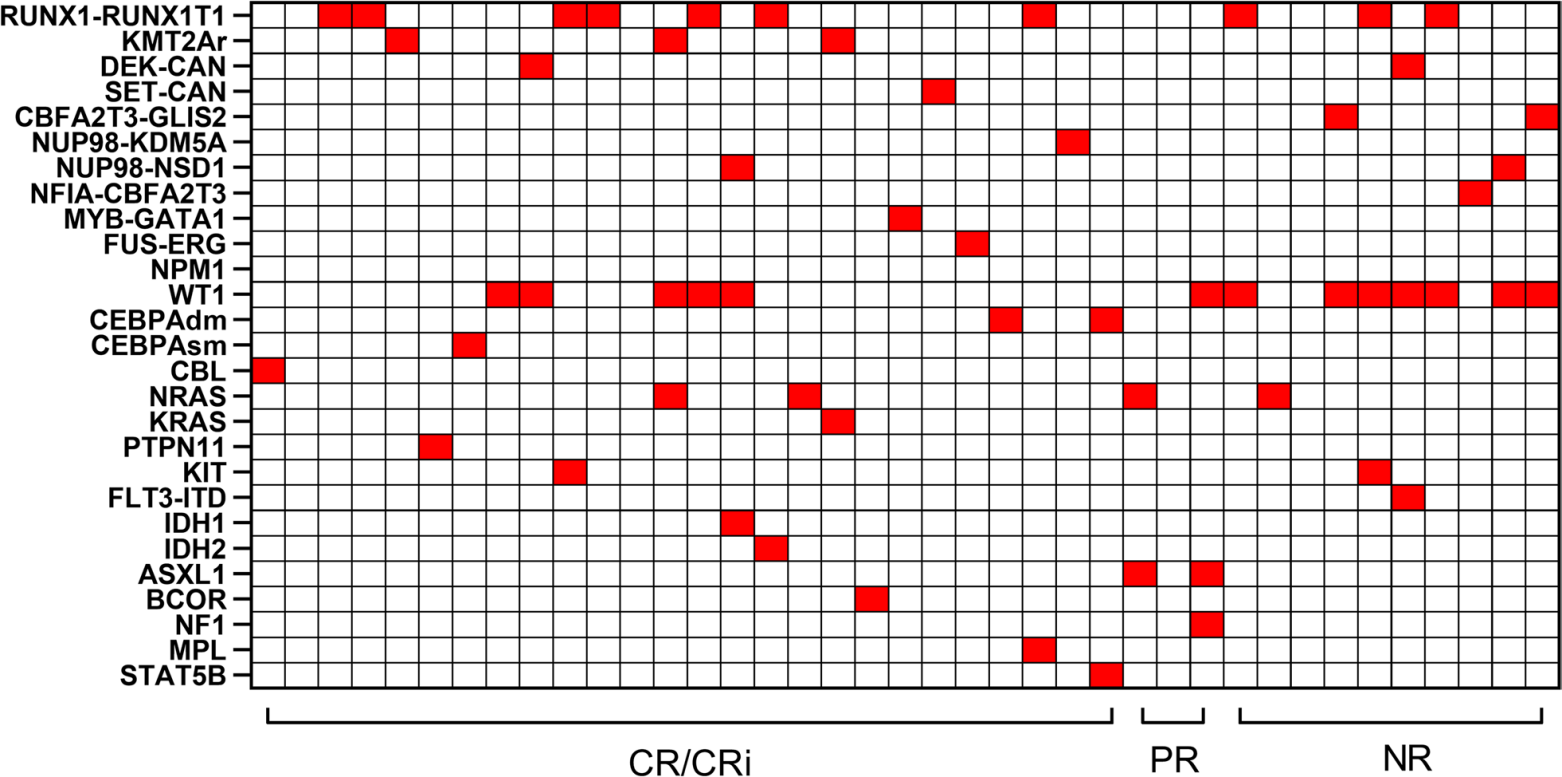
Landscape of gene fusions and mutations in patients with R/R AML. Three groups were divided whether the patient achieved a CR/CRi, PR, or NR. Each row represents a gene, and each column represents a participant. *CEBPAdm* CEBPA double mutation, *CEBPAsm* CEBPA single mutation

Gene detection was performed in all patients. Fusion genes were found in 24 (61.54%) patients, of which RUNX1/RUNX1T1 (25.64%) and KMT2Ar (7.69%) were the most frequently seen. One mutation was seen in 15 (38.46%) patients, and two mutations were found in three (7.69%) patients. As shown in Fig. 2, the most frequently mutated genes were WT1 (33.33%), followed by NRAS (10.26%), CEBPA double mutation (5.13%), ASXL1 (5.13%), IDH1/2 (5.13%), KIT (5.13%), and FLT3-ITD (2.56%). RNA sequencing was performed in four patients of CLAG group and 15 patients of DAC-CLAG group (*P* < 0.001). One patient with CBFA2T3/GLIS2 detected by RNA sequencing was upgraded from intermediate risk to high risk in DAC-CLAG group. According to RNA sequencing, rare fusion gene was found in our study, including CBFA2T3-GLIS2 (5.13%), NUP98-NSD1 (5.13%), NUP98-KDM5A (2.56%), FUS-ERG (2.56%), NFIA-CBFA2T3 (2.56%), and MYB-GATA1 (2.56%) (Fig. 2).

### CR rate after reinduction

Patients received a median of 2 (range 1–3) courses of CLAG therapy, and a median of 1 (range 1–2) course of DAC-CLAG therapy. One patient in the CLAG cohort had an early death due to disease progression and severe infection on day 5. The overall CR/CRi rate was 66.67% for one course and 71.79% for two courses in the whole group. CR/CRi were achieved in 14 (63.64%) CLAG patients and in 12 (70.59%) DAC-CLAG patients after one course (*P* = 0.740). Three patients (13.64%) achieved PR, and five (22.73%) had NR in the CLAG group. By contrast, PR and NR were observed in 0 and 29.41% (5/17) in the DAC-CLAG group. Following the second course, the CR/CRi rate was 72.73% (16/22) with CLAG and 70.59% (12/17) with DAC-CLAG (*P* = 1.000). Molecular MRD negativity at the end of induction was 29.41% (5/17) in CLAG *vs* 43.75% (7/16) in DAC-CLAG patients (*P* = 0.481). FCM MRD negativity was 52.94% in nine of 17 patients treated with DAC-CLAG. While FCM MRD was only performed in two cases in CLAG group.

### Risk factors influencing the efficacy of reinduction

Compared with FAB subsets, M4 patients achieved the highest CR/CRi rate of 81.82%, followed by M5 (58.33%) and M2 (58.33%) patients. Among the individual molecular subgroups, CR/CRi was 70.00% in patients with RUNX1-RUNX1T1, 50.00% in those with NRAS, 53.85% in those with adverse cytogenetic risk, and 38.46% in those with WT1 (Additional file 1: Table S1; Table 2). Response rates in those with other molecular abnormalities are shown in Additional file 1: Table S1. Patients who relapsed after allo-SCT had a lower likelihood of achieving remission again, with a CR rate of 50.00%, compared to 68.57% after non allo-SCT. Patients with rarely seen ASXL1 or CBFA2T3-GLIS2 (both 5.13%) showed NR following the CLAG regimen. Univariate analysis revealed that receiving previous CLAG therapy (*P* = 0.099) and poor cytogenetic risk (*P* = 0.029) were predictive markers of lower CR achievement (Table 2).

**Table 2:** Response in children with R/R AML treated with the CLAG regimen with and without decitabine

Features	Total CR/CRi, % (*n*)	*P* value	CLAG CR/CRi, % (*n*)	DAC-CLAG CR/CRi, % (*n*)
Total	66.67 (26/39)		63.64 (14/22)	70.59 (12/17)
Age				
< 10 years	65.38 (17/26)	1.000	64.29 (9/14)	66.67 (8/12)
≥ 10 years	69.23 (9/13)		62.50 (5/8)	80.00 (4/5)
FAB classification		0.709^a^		
M0	100 (1/1)		− (0/0)	100 (1/1)
M2	58.33 (7/12)		57.14 (4/7)	60.00 (3/5)
M4	81.82 (9/11)		85.71 (6/7)	75.00 (3/4)
M5	58.33 (7/12)		50.00 (3/6)	66.67 (4/6)
M7	100 (1/1)		− (0/0)	100 (1/1)
Myeloid sarcoma	50.00 (1/2)		50.00 (1/2)	− (0/0)
Cytogenetic risk		0.029		
High	53.85 (14/26)		46.15 (6/13)	61.54 (8/13)
Favorable/intermediate	92.31 (12/13)		88.89 (8/9)	100 (4/4)
Karyotype		0.508		
Complex karyotype	60.00 (9/15)		55.56 (5/9)	66.67 (4/6)
Noncomplex karyotype	70.83 (17/24)		69.23 (9/13)	72.73 (8/11)
Enroll after allo-SCT		0.589		
After SCT	50.00 (2/4)		66.67 (2/3)	0 (0/1)
Without SCT	68.57 (24/35)		63.16 (12/19)	75.00 (12/16)
Previous CLAG		0.099		
Yes	25.00 (1/4)		0 (0/1)	33.33 (1/3)
No	71.43 (25/35)		66.67 (14/21)	78.57 (11/14)

*Allo-SCT* allogeneic stem cell transplantation, *CR/CRi* complete remission/CR with incomplete hematopoietic recovery, *CLAG* cladribine, cytarabine, and granulocyte colony-stimulating factor, *DAC* decitabine, *n* number, *FAB* French-American-British, *R/R AML* relapsed or refractory acute myeloid leukemia

^a^Analysis with M2, M4 + M5

### Risk of long-term survival

**Fig. 3:**
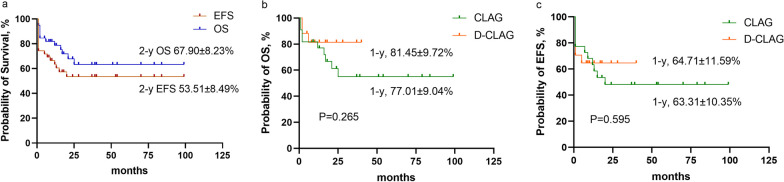
Survival of children with relapsed/refractory acute myeloid leukemia (R/R AML). **a** The 2-year EFS and OS in pediatric R/R AML. **b** The 1-year OS of patients treated with the CLAG or DAC-CLAG regimen. **c** The 1-year EFS of patients treated with the CLAG or DAC-CLAG regimen

Among all 39 patients, 27 (69.23%) subsequently received SCT, while 12 did not. In CLAG group, nine patients received haplo-SCT, one received matched unrelated donor SCT, one received matched sibling donor (MSD) SCT, one received umbilical cord blood transplantation (UCBT), and two received autologous SCT (auto-SCT). In DAC-CLAG group, five cases received matched unrelated donor SCT, and eight received haplo-SCT. In total, 13 patients (76.47%) in the DAC-CLAG cohort and 14 patients (63.64%) in the CLAG cohort subsequently underwent SCT (*P* = 0.494). There was no difference in patients who had auto-SCT or allo-SCT between two groups (*P* = 0.482). Twenty-five children received SCT with CR status, one with PR status, and one with NR. The 1-year, 2-year, and 3-year OS were 78.74% ± 6.72%, 67.90% ± 8.23%, and 63.37 ± 8.84% for the entire group (Fig. 3a). When comparing each group, there was no significant difference between the DAC-CLAG and CLAG regimens, with a 1-year OS of 81.45% ± 9.72% and 77.01% ± 9.04%, respectively (hazard ratio [HR] 0.49, 95% CI 0.16–1.56, *P* = 0.265, Fig. 3b). The 1-year, 2-year, and 3-year EFS were 63.54% ± 7.81%, 53.51% ± 8.49%, and 53.51% ± 8.49% in the whole group (Fig. 3a), with a 1-year EFS of 64.71% ± 11.59% in the DAC-CLAG group and 63.31% ± 10.35% in the CLAG group (HR 0.78, 95% CI 0.30–2.05, *P* = 0.595, Fig. 3c).

**Fig. 4:**
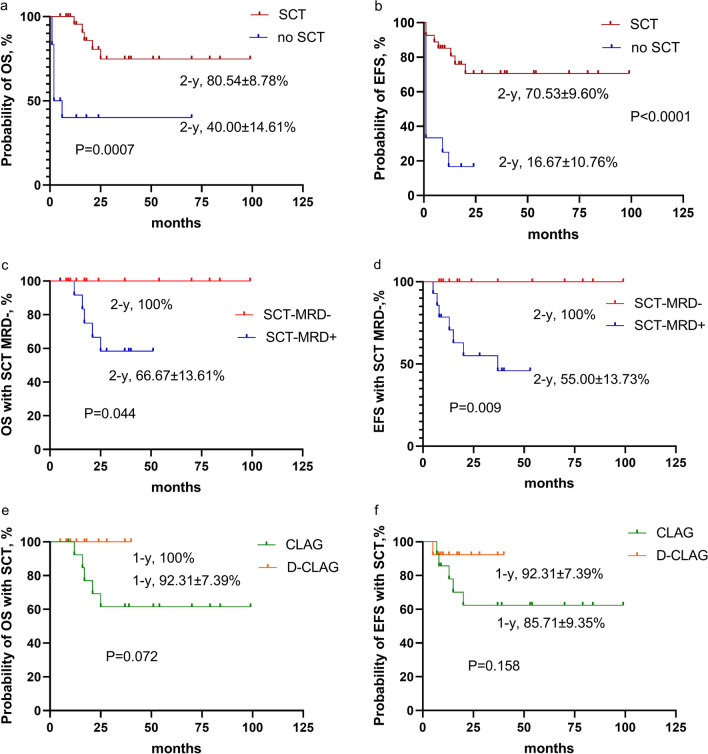
OS and EFS of children with relapsed/refractory acute myeloid leukemia (R/R AML) who underwent SCT. **a** The 2-year OS in patients with or without subsequent SCT. **b** The 2-year EFS in patients with or without subsequent SCT. **c** The 2-year OS of patients who underwent SCT and were molecular MRD negative or positive. **d** The 2-year EFS of patients who underwent SCT and were molecular MRD negative or positive. **e** The 1-year OS of patients who underwent SCT was higher in the DAC-CLAG cohort than in the CLAG cohort. **f** The 1-year EFS of patients who underwent SCT was higher in the DAC-CLAG cohort than in the CLAG cohort

SCT was associated with a significant improvement in OS and EFS compared with no SCT (2-year OS 80.54% ± 8.78% vs 40.00% ± 14.61%; 2-year EFS 70.53% ± 9.60% vs 16.67% ± 10.76%; *P* = 0.0007 and *P* < 0.0001) (Fig. 4a, b). Of the 27 patients who received SCT, 14 patients were molecular MRD positive, while 13 patients with molecular MRD negative. All MRD negative patients remained in remission and were alive at the last follow-up day, while MRD positive patients had a lower 2-year OS of 66.67% ± 13.61% and EFS of 55.00% ± 13.73% *P* = 0.044; *P* = 0.009; Fig. 4c, d). The 1-year relapse rate after SCT was 7.69% (1/13) and 28.57% (4/14) in DAC-CLAG and CLAG group (*P* = 0.326), respectively. The 1-year accumulative relapse rate after SCT was 7.69% ± 7.39% in DAC-CLAG and 29.87% ± 12.57% in CLAG group (*P* = 0.129). All the events after SCT were related with the relapses. The 1-year OS and EFS after SCT were higher in the DAC-CLAG than in the CLAG cohort (100% *vs* 92.31% ± 7.39%,* P* = 0.072; 92.31% ± 7.39% and 85.71% ± 9.35%, *P* = 0.158; Fig. 4e, f). Other risks for prognosis are shown in Additional file 1: Table S2.

Univariate analysis revealed that favorable prognosis was related to good cytogenetics (HR = 0.160, 95% CI 0.05–0.52, *P* = 0.002), non-complex karyotype (HR = 0.35, 95% CI 0.11–1.18; *P* = 0.056), CR on reinduction (HR = 0.11, 95% CI 0.03–0.40; *P* < 0.0001) and bridging to SCT (HR = 0.18, 95% CI 0.05–0.75; *P* = 0.0007). Further Cox regression model analysis showed that the use of a HMA (*P* = 0.049), and bridging to SCT (*P* = 0.011) were independent good prognostic factors for OS.

### Adverse effects

Each patient received 1–3 courses of induction therapy with a total of 59 courses in the entire population. The most common adverse effects were grade 3–4 hematologic toxicity and febrile neutropenia (Table 3). Myelosuppression was common. Thrombocytopenia, neutropenia, and anemia were observed in all cases. The recovery times for neutropenia (> 0.5 × 10^9^/L) and thrombocytopenia (> 20 × 10^9^/L) were longer in the DAC-CLAG group than in the CLAG group (median 20 and 15 days, *P* = 0.0004; median 16 and 13 days, *P* = 0.042). The most common infections were pneumonia (42.37%), intestinal infection (22.03%), soft-tissue infection (15.25%) and central venous catheter (CVC)-related infection (11.86%). The most common pathogens were bacteria and fungi. There was no difference in the incidence of infection between the two groups (Table 3). No severe bleeding was observed. Significant cardiac, hepatic, or renal toxicities were not noted in the groups. One death occurred in the CLAG group during chemotherapy administration. No infection-related deaths were recorded in the DAC-CLAG group.

**Table 3:** Adverse effects of salvage chemotherapy with the CLAG regimen with or without decitabine in R/R AML

Adverse effects	CLAG (cycle = 39)	DAC-CLAG (cycle = 20)	*P* value
Hematologic toxicity ≥ grade 3, *n* (%)	38 (97.44)	20 (100)	1.000
Neutropenia recovery ≥ 0.5 × 10^9^/L, days, (range)	15 (0^a^–35)	20 (13–44)	0.0004
Thrombocytopenia recovery ≥ 20 × 10^9^/L, days, (range)	13 (0^a^–35)	16 (10–33)	0.042
Infections			
Septic shock, *n* (%)	3 (7.69)	2 (10.00)	1.000
Pneumonia, *n* (%)	16 (41.03)	9 (45.00)	0.788
Mechanical ventilation required, *n* (%)	1 (2.56)	1 (5.00)	1.000
Diarrhea or intestinal infection, n (%)	8 (20.51)	5 (25.00)	0.746
Skin/soft-tissue infection, *n* (%)	6 (15.38)	3 (15.00)	1.000
CVC, *n* (%)	5 (12.82)	2 (10.00)	1.000
Perianal inflammation, *n* (%)	3 (7.69)	2 (10.00)	1.000
Fungi identified, n (%)	*Candida albicans*, 3 (7.69) Aspergillus, 2 (5.13)	*Candida albicans*, 1 (5.00) Candida parapsilosis, 1 (5.00) Aspergillus, 1 (5.00)	1.000
Bacteria identified, *n* (%)	12 (30.77)	8 (40.00)	0.566
Bacteremia, *n* (%)	5 (12.82)	1 (5.00)	0.653
Covid-19, identified, *n* (%)	0	2 (10.00)	0.111
Die during the course, *n* (%)	1 (2.56)	0	1.000

*CLAG* cladribine, cytarabine, and granulocyte colony-stimulating factor, *CVC* central venous catheter, *DAC* decitabine, *n* number, *R/R AML* relapsed or refractory acute myeloid leukemia

^a^Neutropenia or thrombocytopenia not less than 0.5 × 10^9^/L or 20 × 10^9^/L during the cycles

## Discussion

Despite modern, intensified chemotherapeutic regimens and enhanced supportive care, children with R/R AML still have poor outcomes, and new treatment protocols are urgently needed. Conventional intensive regimens contain either “7 + 3” based regimens with “X” or M, etoposide, and high-dose cytarabine (MEC), which are often ineffective in R/R AML. Therefore, developing new therapeutic options to achieve CR is necessary to proceed to subsequent SCT.

In this study, we administered DAC as HMA priming of the CLAG salvage regimen, which exhibited significant anti-leukemia activity in AML with a CR/CRi rate of 70.6% compared to 63.6% for the CLAG regimen. The CLAG ± DAC regimen showed a significantly higher CR rate than the conventional regimen (22–23%) in children [1, 2]. There are no data on the CR rate for DAC combined with cladribine-based regimens in children. In adults, a CR of 65% in R/R AML and 85% in newly diagnosed AML with the DAC-CLAG + M regimen was reported [14]. Other regimens such as cladribine plus low-dose cytarabine alternating with DAC in older patients with newly diagnosed AML was observed to have a CR/CRi rate of 68% [15]. Cladribine-based regimens such as CLAG ± M without DAC can also induce remission in more than half (50–70%) of adult patients [3, 16] and a CR rate of 67–75% in children [1, 2].

Improved outcomes have been reported with the prevalent use of purine analog combinations during reinduction. Cladribine, a purine analog, has been found to have activity against acute leukemias in adults as well as children over the past 30 years. As a single agent [17, 18] and in combination with cytarabine [19, 20], topotecan [21], as well as idarubicin [22], a beneficial trend was recorded in children. Cladribine is usually combined with cytarabine plus “X” in induction regimens nowadays. These regimens which are administered to patients with prior administration of cladribine can increase the cellular uptake of cytarabine and accumulation of cytarabine triphosphate (Ara-CTP), accompanied by the sensitization of leukemic blasts to cytarabine with G-CSF [1, 23].

We also observed a higher proportion of molecular MRD negativity in the DAC-CLAG group compared with the CLAG group (43.8% vs 29.4%), indicating a quick deep remission in these patients. The same result was observed in epigenetic priming induction compared with standard induction in children with newly diagnosed AML [24]. Higher DNA methylation has been observed in AML and promoter hypermethylation was related to poor prognosis [25], showing epigenetic modifications in the pathogenesis of acute leukemia. A study showed that 83% of patients with relapsed AML had higher DNA methylation levels than those with primary AML [11]. The addition of a HMA to reinduction chemotherapy contributed to achieving remission in R/R AML trials [14, 26]. However, as a single agent, it has yielded modest benefit, with remission rates of 17–25% in adults [27, 28] and responses in 3 of 8 patients in a pediatric report [29]. DAC added to the CLAG regimen may exploit the synergy between DAC and cladribine to improve treatment response. Furthermore, chemo-sensitizing effects may be greater if the “priming” schedule of the HMA is used rather than combined with the other chemotherapeutics [30, 31].

In addition, poor responses occurred in patients with the subtype of high-risk cytogenetics, such as CBFA2T3-GLIS2, ASXL1 and WT1. However, data are limited in our study. A relatively high CR rate of RUNX1-RUNX1T1 was expressed in all cytogenetic subsets. Patients with KMT2A rearrangement showed 100% remission in this cohort, two with KMT2A-MLLT11 and one with KMT2A-MLLT10. Previous reports showed it is more difficult to achieve new remission in AML patients who relapse after transplantation, with CR rates of only 10–32% [32, 33]. We found that when using the CLAG-based regimen, the CR rate reached 50%. However, this rate was lower than in those who did not undergo SCT. Additionally, if patients had previously received the CLAG regimen, there was little chance of gaining remission again using CLAG or priming with DAC.

The outcome of R/R AML remains poor. We reported a total 3-year OS of 63.4% and EFS of 53.5% in cladribine-based regimens, which was higher than traditional induction with a 3-year OS of 22.2–40.4% and EFS of 22.2–34.9% [1, 2]. Univariate analysis revealed that poor prognosis was related to poor cytogenetics, failure to achieve CR, and not undergoing SCT in our report. To improve the long-term survival, bridging to transplantation was an effective option for R/R AML patients. However, salvage transplantation with residual blasts was not ideal [26]. In our previous report, 2-year OS increased to 67% in R/R AML children who achieved a second CR before allo-SCT [1]. In the present study, the 2-year OS was much more promising for patients bridging to SCT (80%) than those without SCT (40%), especially in patients who were MRD negative (100%). SCT can both extend OS and EFS significantly. Furthermore, superior OS and EFS were also observed in the DAC-CLAG group with more patients receiving SCT. Cox regression analysis strongly indicated that DAC priming and bridging SCT were related to better OS. The relapse rate after SCT was lower in DAC-CLAG cohort than CLAG. That may relate with higher molecular MRD negativity and HMA administration in DAC-CLAG group.

For cases with R/R disease pre-transplantation, relapse prophylaxis was performed in our study. Early withdrawal of immunosuppression would be implemented carefully as suggested in guideline [34]. Prophylactic donor lymphocyte infusion (DLI) was performed in patients who did not achieve hematological remission or CR3 before transplantation. Preventive application of DAC for all patients stared in 2019. And targeted drug sorafenib was used for patients with FLT3-ITD. In case of MRD turning positive after SCT, chemotherapy prior to DLI, or interferon-α, and/or targeted drugs have been considered [35]. In the state of hematological relapse of AML after transplantation, chemotherapy plus sequential therapeutic DLI was recommended. Targeted drugs or demethylated drugs would be added to the chemotherapy regimen. One patient who relapsed after allo-SCT in CLAG group received a secondary transplantation for the treatment of relapse. However, he had an extramedullary recurrence in central nervous system and scrotum.

Both regimens were well tolerated. Hematologic toxicities, as expected, occurred in nearly all patients. DAC-CLAG was associated with more toxicity such as prolonged cytopenia than CLAG. This is similar to the report of 20 days recovery time for neutropenia and 18 days recovery time for thrombocytopenia with regimens involving DAC, cladribine, idarubicin or homoharringtonine, and cytarabine [26]. The incidence of neutropenic fever and infections was comparable in the two regimens. Septic shock and mechanical ventilation occurred in few patients. There was no trend toward the risk of severe infection in the DAC-treated cohort. Both regimens showed minimal extramedullary toxicity. Only 1 patient died during administration of the CLAG regimen due to disease progression and infection.

## Conclusion

This first-in-pediatrics retrospective study demonstrates that the DAC-CLAG regimen was an appropriate option for patients with R/R AML and had acceptable toxicity. DAC-CLAG resulted in a superior induction response compared to CLAG in pediatrics. The analysis identified subsets of patients with distinct clinical outcomes. The regimen appeared to induce high remission in patients with good cytogenetics such as RUNX1-RUNX1T1. Achieving remission, low cytogenetic risk, and subsequent SCT were associated with good prognosis in R/R AML. It is worth noting that SCT and priming epigenetic modification improved the prognosis of R/R AML as independent factors. Limited patients in the cohort may have influenced the precision of results in the exploratory subgroup analysis and further studies with large data are required.

### Supplementary Information

**Additional file 1**. The CR rate among molecular subtypes and prognosis risks in patients with R/R AML.

## Data Availability

The datasets generated during and/or analyzed during the current study are available from the corresponding author on reasonable request.

## Abbreviations

AML: Acute myeloid leukemia
Auto: Autologous
Allo: Allogeneic
BM: Bone marrow
CR: Complete remission
CRi: CR with incomplete hematopoietic recovery
CLAG: Cladribine, cytarabine, and granulocyte-stimulating factor
CVC: Central venous catheter
DAC: Decitabine
DLI: Donor lymphocyte infusion
ELN: European LeukemiaNet
EFS: Event-free survival
FCM: Flow cytometry
FAB: French–American–British
HMAs: Hypomethylating agents
M: Mitoxantrone
MICM: Morphology, immunology, molecular and cytogenetic
MRD: Minimal residual disease
MSD: Matched sibling donor
NR: No remission
NGS: Next-generation sequencing
OS: Overall survival
PR: Partial remission
RT-PCR: Reverse transcription-polymerase chain reaction
R/R: Relapsed or refractory
SCT: Stem cell transplantation
UCBT: Umbilical cord blood transplantation

## References

[1] Zhang N, Shao JB, Li H, Yang JW, Chen K, Zhu JS (2020). Re-induction with modified CLAG regimen in relapsed or refractory acute myeloid leukemia in children bridging to allogeneic hematopoietic stem cell transplantation. World J Pediatr.

[2] Ruan M, Liu LP, Zhang AL, Quan Qi B, Liu F, Liu TF (2021). Improved outcome of children with relapsed/refractory acute myeloid leukemia by addition of cladribine to re-induction chemotherapy. Cancer Med.

[3] Wrzesien-Kus A, Robak T, Lech-Maranda E, Wierzbowska A, Dmoszynska A, Kowal M (2003). A multicenter, open, non-comparative, phase II study of the combination of cladribine (2-chlorodeoxyadenosine), cytarabine, and G-CSF as induction therapy in refractory acute myeloid leukemia-a report of the Polish Adult Leukemia Group (PALG). Eur J Haematol.

[4] Park H, Youk J, Kim I, Yoon SS, Park S, Lee JO (2016). Comparison of cladribine- and fludarabine-based induction chemotherapy in relapsed or refractory acute myeloid leukaemia. Ann Hematol.

[5] Robak T, Wrzesień-Kuś A, Lech-Marańda E, Kowal M, Dmoszyńska A (2000). Combination regimen of cladribine (2-chlorodeoxyadenosine), cytarabine and G-CSF (CLAG) as induction therapy for patients with relapsed or refractory acute myeloid leukemia. Leuk Lymphoma.

[6] Scheckel CJ, Meyer M, Betcher JA, Al-Kali A, Foran J, Palmer J (2020). Efficacy of mitoxantrone-based salvage therapies in relapsed or refractory acute myeloid leukemia in the Mayo Clinic Cancer Center: analysis of survival after 'CLAG-M' vs 'MEC'. Leuk Res.

[7] Yao H, Zhang C, Tan X, Li J, Yin X, Deng X (2023). Efficacy and toxicity of CLAG combined with pegylated liposomal doxorubicin in the treatment of refractory or relapsed acute myeloid leukemia. Cancer Med.

[8] Wang H, Wang L, Li C, Wuxiao Z, Shao R, Wang H (2020). Cladribine with granulocyte Colony-stimulating factor, Cytarabine, and Aclarubicin regimen in refractory/relapsed acute myeloid leukemia: a phase II multicenter study. Oncologist.

[9] Ma YY, Zhao M, Liu Y, Zhao DF, Wang LX, Chen XP (2019). Use of decitabine for patients with refractory or relapsed acute myeloid leukemia: a systematic review and meta-analysis. Hematology.

[10] Grövdal M, Khan R, Aggerholm A, Antunovic P, Astermark J, Bernell P (2007). Negative effect of DNA hypermethylation on the outcome of intensive chemotherapy in older patients with high-risk myelodysplastic syndromes and acute myeloid leukemia following myelodysplastic syndrome. Clin Cancer Res.

[11] Kroeger H, Jelinek J, Estécio MR, He R, Kondo K, Chung W (2008). Aberrant CpG island methylation in acute myeloid leukemia is accentuated at relapse. Blood.

[12] Spurgeon S, Yu M, Phillips JD, Epner EM (2009). Cladribine: not just another purine analogue?. Expert Opin Investig Drugs.

[13] Döhner H, Estey E, Grimwade D, Amadori S, Appelbaum FR, Büchner T (2017). Diagnosis and management of AML in adults: 2017 ELN recommendations from an international expert panel. Blood.

[14] Palmieri R, Buckley SA, Othus M, Halpern AB, Percival MM, Scott BL (2020). Randomized phase 1 study of sequential (“primed”) vs. concurrent decitabine in combination with cladribine, cytarabine, G-CSF, and mitoxantrone (CLAG-M) in adults with newly diagnosed or relapsed/refractory acute myeloid leukemia (AML) or other high-grade myeloid neoplasm. Leuk Lymphoma.

[15] Kadia TM, Ravandi F, Borthakur G, Konopleva M, DiNardo CD, Daver N (2021). Long-term results of low-intensity chemotherapy with clofarabine or cladribine combined with low-dose cytarabine alternating with decitabine in older patients with newly diagnosed acute myeloid leukemia. Am J Hematol.

[16] Wierzbowska A, Robak T, Pluta A, Wawrzyniak E, Cebula B, Hołowiecki J (2008). Cladribine combined with high doses of arabinoside cytosine, mitoxantrone, and G-CSF (CLAG-M) is a highly effective salvage regimen in patients with refractory and relapsed acute myeloid leukemia of the poor risk: a final report of the Polish Adult Leukemia Group. Eur J Haematol.

[17] Santana VM, Mirro J, Kearns C, Schell MJ, Crom W, Blakley RL (1992). 2-Chlorodeoxyadenosine produces a high rate of complete hematologic remission in relapsed acute myeloid leukemia. J Clin Oncol.

[18] Santana VM, Hurwitz CA, Blakley RL, Crom WR, Luo X, Roberts WM (1994). Complete hematologic remissions induced by 2-chlorodeoxyadenosine in children with newly diagnosed acute myeloid leukemia. Blood.

[19] Rubnitz JE, Razzouk BI, Srivastava DK, Pui CH, Ribeiro RC, Santana VM (2004). Phase II trial of cladribine and cytarabine in relapsed or refractory myeloid malignancies. Leuk Res.

[20] Rubnitz JE, Crews KR, Pounds S, Yang S, Campana D, Gandhi VV (2009). Combination of cladribine and cytarabine is effective for childhood acute myeloid leukemia: results of the St Jude AML97 trial. Leukemia.

[21] Inaba H, Stewart CF, Crews KR, Yang S, Pounds S, Pui CH (2010). Combination of cladribine plus topotecan for recurrent or refractory pediatric acute myeloid leukemia. Cancer.

[22] Chaleff S, Hurwitz CA, Chang M, Dahl G, Alonzo TA, Weinstein H (2012). Phase II study of 2-chlorodeoxyadenosine plus idarubicin for children with acute myeloid leukaemia in first relapse: a paediatric oncology group study. Br J Haematol.

[23] Gandhi V, Estey E, Keating MJ, Chucrallah A, Plunkett W (1996). Chlorodeoxyadenosine and arabinosylcytosine in patients with acute myelogenous leukemia: pharmacokinetic, pharmacodynamic, and molecular interactions. Blood.

[24] Gore L, Triche TJ, Farrar JE, Wai D, Legendre C, Gooden GC (2017). A multicenter, randomized study of decitabine as epigenetic priming with induction chemotherapy in children with AML. Clin Epigenetics.

[25] Shimamoto T, Ohyashiki JH, Ohyashiki K (2005). Methylation of p15(INK4b) and E-cadherin genes is independently correlated with poor prognosis in acute myeloid leukemia. Leuk Res.

[26] Hui Y, Li Y, Tong X, Huang L, Mao X, Huang L (2021). Reinduction chemotherapy regimen involved decitabine and cladribine improves the prognosis of patients with relapsed or refractory acute myeloid leukemia: a preliminary study. Int J Cancer.

[27] Al-Ali HK, Jaekel N, Junghanss C, Maschmeyer G, Krahl R, Cross M (2012). Azacitidine in patients with acute myeloid leukemia medically unfit for or resistant to chemotherapy: a multicenter phase I/II study. Leuk Lymphoma.

[28] Maurillo L, Venditti A, Spagnoli A, Gaidano G, Ferrero D, Oliva E (2012). Azacitidine for the treatment of patients with acute myeloid leukemia: report of 82 patients enrolled in an Italian compassionate program. Cancer.

[29] Phillips CL, Davies SM, McMasters R, Absalon M, O'Brien M, Mo J (2013). Low dose decitabine in very high risk relapsed or refractory acute myeloid leukaemia in children and young adults. Br J Haematol.

[30] Hollenbach PW, Nguyen AN, Brady H, Williams M, Ning Y, Richard N (2010). A comparison of azacitidine and decitabine activities in acute myeloid leukemia cell lines. PLoS ONE.

[31] Leonard SM, Perry T, Woodman CB, Kearns P (2014). Sequential treatment with cytarabine and decitabine has an increased antileukemia effect compared to cytarabine alone in xenograft models of childhood acute myeloid leukemia. PLoS ONE.

[32] Schmid C, Labopin M, Nagler A, Niederwieser D, Castagna L, Tabrizi R (2012). Treatment, risk factors, and outcome of adults with relapsed AML after reduced intensity conditioning for allogeneic stem cell transplantation. Blood.

[33] Mielcarek M, Storer BE, Flowers ME, Storb R, Sandmaier BM, Martin PJ (2007). Outcomes among patients with recurrent high-risk hematologic malignancies after allogeneic hematopoietic cell transplantation. Biol Blood Marrow Transplant.

[34] Wang Y, Chen H, Chen J, Han M, Hu J (2018). The consensus on the monitoring, treatment, and prevention of leukemia relapse after allogeneic hematopoietic stem cell transplantation in China. Cancer Lett.

[35] Tarlock K, Sulis ML, Chewning JH, Pollard JA, Cooper T, Gamis A (2022). Hematopoietic cell transplantation in the treatment of pediatric acute myelogenous leukemia and myelodysplastic syndromes: guidelines from the american society of transplantation and cellular therapy. Transplant Cell Ther.

