# Supplementary material for: Decitabine as epigenetic priming with CLAG induce improved outcome of relapsed or refractory acute myeloid leukemia in children
Source: Clin Epigenetics. 2024 May 9;16:63. doi: 10.1186/s13148-024-01677-z (PMC11080195; doi:10.1186/s13148-024-01677-z)
Supplement: Supplementary file 1 — Additional file 1. The CR rate among molecular subtypes and prognosis risks in patients with R/R AML. [file 13148_2024_1677_MOESM1_ESM.docx]

**Table S1 The CR rate in patients with R/R AML treated with the CLAG regimen with and without decitabine among molecular subtypes.**

| Cytogenetic features | Total CR, % (n) | CLAG CR, % (*n*) | DAC-CLAG CR, % (*n*) |
| --- | --- | --- | --- |
| t(8;21)/ RUNX1/RUNX1T1 | 70.00 (7/10) | 71.43 (5/7) | 66.67 (2/3) |
| WT1 | 38.46 (5/13) | 50.00 (4/8) | 20.00 (1/5) |
| NRAS | 50.00 (2/4) | 33.33 (1/3) | 100 (1/1) |
| KMT2Ar | 100 (3/3) | 100 (2/2) | 100 (1/1) |
| Nup98r | 66.67 (2/3) | - (0/0) | 66.67 (2/3) |
| DEK/CAN or SET/CAN | 66.67(2/3) | 100 (1/1) | 50.00 (1/2) |
| IDH1/2 | 100 (2/2) | - (0/0) | 100 (2/2) |
| KIT | 50.00 (1/2） | 50.00 (1/2) | - (0/0) |
| CBFA2T3-GLIS2 | 0 (0/2) | 0 (0/1) | 0 (0/1) |
| ASXL1 | 0 (0/2) | 0 (0/2) | - (0/0) |
| CEBPA dm | 100 (2/2) | - (0/0) | 100 (2/2) |
| FLT3-ITD | 0 (0/1) | - (0/0) | 0 (0/1) |

**Table S2 Univariate analysis of prognosis risks in R/R-AML patients treated with CLAG with or without decitabine**.

| Characteristics | 2-year EFS, % | *P* value | 2-year OS, % | *P* value |
| --- | --- | --- | --- | --- |
| Total | 53.51±8.49 |  | 67.90%±8.23 |  |
| Karyotype |  | 0.079 |  | 0.056 |
| Complex | 32.00±14.01 |  | 41.91±15.19 |  |
| No complex | 65.42±9.96 |  | 82.45±8.01 |  |
| Cytogenetic risk |  | 0.002 |  | 0.002 |
| Adverse | 33.04±10.18 |  | 49.06±11.44 |  |
| Favorable/ intermediate | 92.31±7.39 |  | 100 |  |
| Response at reinduction |  | <0.0001 |  | <0.0001 |
| CR/CRi | 77.43±9.14 |  | 84.21±8.45 |  |
| PR/NR | 7.69±7.39 |  | 33.66±14.38 |  |
| Induction regimens |  | 0.595 |  | 0.265 |
| DAC-CLAG | 64.71±11.59 |  | 81.45±9.72 |  |
| CLAG | 48.21±10.97 |  | 61.18±10.88 |  |
| Bridging to SCT |  |  |  |  |
| No SCT | 16.67±10.76 | <0.0001 | 40.00±14.61 | 0.0007 |
| With SCT | 70.53±9.60 |  | 80.54±8.78 |  |
| SCT with MRD- | 100 | 0.009 | 100 | 0.044 |
| SCT with MRD+ | 55.00±13.73 |  | 66.67±13.61 |  |

MRD- defined as molecular MRD negative.
